# Genome-wide identification of the *GRAS* gene family and evidence for the involvement of *PgGRAS48* in main root development in *Panax ginseng*

**DOI:** 10.3389/fpls.2025.1603268

**Published:** 2025-06-13

**Authors:** Yihan Wang, Ping Wang, Peng Di, Yingping Wang

**Affiliations:** State Local Joint Engineering Research Center of Ginseng Breeding and Application, Jilin Agricultural University, Changchun, China

**Keywords:** GRAS gene family, *Panax ginseng*, expression pattern analysis, main root, GA

## Abstract

*Panax ginseng* C. A. Meyer (ginseng) is one of the most widely used traditional Chinese medicinal herbs, with its roots as the primary medicinal part garnering significant attention due to their therapeutic potential. The *GRAS* [*GRI* (*Gibberellic Acid Insensitive*), *RGA* (*Repressor of GAI-3 mutant*), and *SCR* (*Scarecrow*)] genes are a class of widely distributed plant-specific transcription factors that play crucial roles in various physiological processes including root formation, fruit development, hormone signaling, and stem cell maintenance. This study systematically identified 139 *GRAS* genes (*PgGRAS*) in the ginseng genome for the first time, analyzing their complexity and diversity through protein domain structure, phylogenetic relationships, gene structure, and cis-acting element prediction. Evolutionary analysis revealed that all *PgGRAS* members were divided into 14 evolutionary branches, including a novel species-specific subfamily PG28, with segmental duplication being the primary driver of family expansion. RNA-seq analysis uncovered tissue-specific expression patterns of the *PgGRAS* gene family. qRT-PCR validation demonstrated that *PgGRAS48*, a member of the SCL3 subfamily, was significantly highly expressed in the main root and upregulated upon GA treatment, suggesting its potential regulatory role in main root development. Therefore, this gene was selected for further investigation. Overexpression of *PgGRAS48* significantly increased the main root length in *Arabidopsis thaliana* (*A. thaliana*), accompanied by elevated endogenous GA levels. Subcellular localization, molecular docking, Bimolecular Fluorescence Complementation (BIFC) and yeast two-hybrid (Y2H) experiments confirmed the interaction between *PgGRAS48* (SCL3) and *PgGRAS2* (DELLA) in the nucleus, revealing the molecular mechanism by which SCL3-DELLA regulates main root elongation through gibberellin (GA) biosynthesis or signaling pathways. This study elucidates the molecular network of the *GRAS* family in root development in ginseng, providing key targets for the targeted improvement of root architecture in medicinal plants.

## 1 Introduction

The *GRAS* gene family is a group of plant-specific transcription regulators, named after the first three identified members: *GAI* (*gibberellic acid insensitive*), *RGA* (*repressor of GA1–3 mutant*), and *SCR* (*scarecrow*) (Di Laurenzio et al., 1996; Peng et al., 1997; Silverstone et al., 1998). Members of this family are widely distributed across the plant kingdom, with typical GRAS proteins consisting of 400–770 amino acids and exhibiting distinct structural features (Waseem et al., 2022). The C-terminal region contains five highly conserved domains: LHRI, VHIID, LHRII, PFYRE, and SAW (Hakoshima, 2018). Studies have shown that VHIID, as the core element, assembles with the flanking LHR I/II regions into a composite structure. This structure, formed by approximately 100 amino acids with atypical 7-amino acid repeat units, mediates DNA binding and protein-protein interactions (Hakoshima, 2018). The C-terminal LHR I region contains a putative nuclear localization signal capable of recognizing SV40-type nuclear localization sequences (Bolle, 2004; Raikhel, 1992). The PFYRE motif is less conserved than VHIID, but the potential phosphorylation of its tyrosine residues suggests its involvement in post-translational regulation (Pysh et al., 1999). Although the exact function of the SAW motif remains unclear, the high conservation of its amino acid residues during evolution indicates its potential role in maintaining protein structural stability or regulating functional activity (Bolle, 2004; Sun et al., 2011). In the N-terminal region, GRAS proteins exhibit significant structural plasticity: this region is highly variable and intrinsically disordered regions (IDRs), with conformational flexibility enabling dynamic binding to different ligands (Sun et al., 2012). When interacting with target proteins, these disordered regions can form specific molecular recognition interfaces, thereby facilitating functional differentiation in processes such as gibberellin (GA) signaling, photomorphogenesis, and meristem regulation (Waseem et al., 2022).

In model plants such as *A. thaliana* and *Oryza sativa* (*O. sativa*), 33 and 60 *GRAS* family members have been identified, respectively. These *GRAS* members are classified into 13 subfamilies: LISCL, LAS, SCL4/7, DLT, SCL3, DELLA, SCR, HAM, SHR, PAT1, Os43, Os4, and Os3 (Tian et al., 2004). Due to significant differences in amino acid sequences among subfamilies, it is speculated that each subfamily may perform unique functions. SCL3 (SCARECROW-LIKE 3) is a key member of the *GRAS* transcription factor family, regulating the GA signaling pathway by antagonizing DELLA proteins (core repressors of GA signaling), thereby promoting main root elongation and lateral root development (Zhang et al., 2011). Its specific expression in the endodermis of *A. thaliana* is regulated by the SHR/SCR complex, with the three cooperatively driving cell divisions and differentiation in the root apical meristem (Montiel, 2004). Recent studies have shown that SCL3 not only maintains GA homeostasis by regulating GA biosynthesis genes (such as *GA20ox* and *GA3ox*) but may also be regulated by phosphorylation: the phosphatase CPL3 interacts with DELLA and enhances its stability, suggesting that SCL3 may fine-tune GA signaling dynamics in coordination with post-translational modifiers (Feng et al., 2008; Li et al., 2024). DELLA proteins, as key regulatory elements in the GA signaling pathway, act as negative regulators, inhibiting plant growth and development (De et al., 2008). In *O. sativa*, SLR1 (SLENDER RICE 1) is a homolog of DELLA proteins, and its loss of function leads to excessive elongation of the plant (Itoh, 2002); in *Hordeum*, the SLN1 (SLENDER1) gene encodes a DELLA protein, whose loss of function similarly causes excessive elongation, while its gain of function results in dwarfism (Chandler, 2002). Studies have shown that GA can bind to the gibberellin receptor protein GID1 (gibberellin insensitive dwarf 1) to form a GA/GID1 complex, which then interacts with the DELLA domain at the N-terminus of DELLA proteins to form a new GA/GID1/DELLA ternary complex. Subsequently, the 26S proteasome ubiquitinates and degrades DELLA proteins, thereby relieving their inhibitory effect on plant growth (Hirano et al., 2008). In addition, other subfamilies of the *GRAS* gene family exhibit significant functional diversity across different plant tissues and species. For example, members of the LAS subfamily play a crucial regulatory role in axillary bud growth and development (Grimplet et al., 2016); PAT1 is involved in phytochrome signaling in *A. thaliana* (Wang et al., 2018); HAM maintains the differentiation of the shoot apical meristem by keeping stem cells in an undifferentiated state, enabling continuous differentiation of the apical meristem (Mélanie et al., 2017; Koizumi et al., 2012); both SCR and SHR act as positive regulators of radial root growth in *A. thaliana* and play an important role in the formation of root cell layers (Mélanie et al., 2017). To date, *GRAS* genes have been identified and functionally characterized in a range of field crops and economic crops (Laskar et al., 2021; Liu et al., 2019; Lv et al., 2021).

Ginseng is a perennial herbaceous medicinal plant belonging to the *Araliaceae* family. Its roots are rich in bioactive compounds such as ginsenosides, which possess pharmacological properties including immune regulation and anti-aging (Laskar et al., 2021), making it widely used in medicine and health products. Members of the *GRAS* family are involved in various physiological processes, with subfamilies such as SCL3, SHR, SCR, and LISCL extensively regulating root development, including main root elongation, lateral root formation, and maintenance of the root apical meristem. Therefore, systematic identification of *GRAS* genes in ginseng is crucial for elucidating the molecular mechanisms underlying root development in this medicinal plant.

In this study, we systematically identified 139 *GRAS* transcription factors (*PgGRAS*) from the ginseng genome for the first time, classifying them into 14 evolutionary branches, including a newly discovered species-specific subfamily, PG28. Through chromosomal localization, gene structure analysis, conserved domain identification, and collinearity analysis, we revealed the expansion mechanisms and functional differentiation of these genes. Furthermore, we analyzed the expression patterns of *PgGRAS* genes in different tissues and under various hormone treatments, identifying *PgGRAS48* as a gene associated with root development. Preliminary functional validation and mechanistic exploration were conducted using qRT-PCR, subcellular localization, Y2H, BIFC, and overexpression in transgenic plants. This study aims to systematically identify members of the ginseng *GRAS* gene family through whole-genome analysis, elucidate their evolutionary relationships, structural characteristics, and tissue-specific expression patterns; combine hormone treatment experiments to screen key genes for root development, and delve into the molecular mechanisms by which *PgGRAS48* regulates main root elongation via the gibberellin (GA) signaling pathway, providing theoretical basis and critical targets for genetic improvement of ginseng root architecture.

## 2 Materials and methods

### 2.1 Identification and analysis of the *GRAS* gene family in ginseng

The genome and protein data of ginseng were obtained from the National Genomics Data Center (NGDC, https://ngdc.cncb.ac.cn/) (Wang et al., 2022), and the Hidden Markov Model (HMM) profile of the *GRAS* gene (PF03195) was downloaded from the Pfam database (http://pfam.xfam.org/). Using HMMER 3.2.1 software, sequences matching the structural characteristics (E-value ≤ 1×10^-5^) were screened from the ginseng genome. Sequences with complete GRAS domains were further filtered using the NCBI Conserved Domain Database (https://www.ncbi.nlm.nih.gov/cdd/) and the SMART data-base (http://smart.embl-heidelberg.de/) and confirmed as members of the *GRAS* gene family in ginseng. Subcellular localization was predicted using the WoLF PSORT tool (https://www.genscript.com/wolf-psort.html), and the physicochemical properties of the proteins were analyzed using the ExPASy ProtParam tool (https://web.expasy.org/protparam).

### 2.2 Phylogenetic analysis of the *PgGRAS* gene family

The GRAS protein sequences of *A. thaliana* were obtained from the *A. thaliana* resource database TAIR (http://www.arabidopsis.org/home.html), while the *O. sativa* genome data were sourced from Phytozome v13 (https://phytozome-next.jgi.doe.gov/). These sequences, along with the *GRA*S family members from ginseng, were subjected to multiple sequence alignment using MAFFT (http://mafft.cbrc.jp/alignment/software/). A maximum likelihood phylogenetic tree was constructed using IQ-TREE based on the JTTDCMut+F+R4 model, with branch support assessed through 1000 bootstrap replicates. The resulting phylogenetic tree was visualized and annotated using iTOL (https://itol.embl.de/).

### 2.3 Structural, conserved domain, and cis-acting element analysis of the *PgGRAS* gene family

Using the MEME online tool (https://meme-suite.org/meme/doc/meme.html) with the following parameters: maximum number of motifs = 20, motif width range = 10–100 amino acids, and E-value threshold ≤ 1×10^^-^5^, to identify conserved motifs in PgGRAS proteins. Based on the GFF3 annotation file (BioProject: PRJCA006678; Accession: GWHBEIL00000000.1) of the ginseng genome (Wang et al., 2022), analyze the exon-intron structure of *PgGRAS* genes. Extract the 1500 bp upstream sequences of the transcription start sites (TSS) of *PgGRAS* genes from the ginseng genome and predict cis-regulatory elements using the PlantCARE database (http://bioinformatics.psb.ugent.be/webtools/plantcare/html/).

### 2.4 Replication and synteny analysis of *PgGRAS g*enes

Obtain the genome data of *Daucus carota* from Phytozome v13 (https://phytozome-next.jgi.doe.gov/) and the genome data of *Panax quinquefolium* and *Panax stipuleanatus* from the National Genomics Data Center (https://ngdc.cncb.ac.cn/). Perform intra- and inter-species synteny analysis using MCScanX (E-value ≤ 1×10^-5^) to identify orthologous and paralogous relationships. Calculate the non-synonymous substitution rate (Ka) and synonymous substitution rate (Ks) using KaKs_Calculator 2.0 and evaluate selection pressure through the Ka/Ks ratio.

### 2.5 RNA sequencing and expression analysis

Obtain RNA-seq data of different tissue parts of ginseng from the NCBI database (Accession Number: PRJNA302556) and analyze the expression data under different hormone treatments. Preprocess and normalize the raw data to obtain TPM values and use R 4.3.1 (https://www.r-project.org) to draw the heatmap of *PgGRAS* gene expression.

### 2.6 RNA extraction and qRT-PCR analysis

RNA extraction and qRT-PCR analysis were performed using tissues from 3-year-old ginseng plants (for tissue-specific expression) or 5-week-old seedlings (for hormone treatments). For tissue-specific analysis, three independent biological replicates were collected from each tissue type (main root periderm, cortex, stele, lateral roots, fibrous roots, rhizomes). For hormone treatments, seedlings with three true leaves were sprayed with ABA (50 μM), IAA (10 μM), 6-BA (75 μM), GA3 (100 μM), or distilled water (control), and samples were harvested after 5 hours with three biological replicates per treatment group. Total RNA was extracted by RNAprep pure plant kit (TIANGEN, Beijing, China) and reverse transcribed into cDNA.

Gene-specific primers were designed using Primer Premier 5 (**Supplementary Table S1**) and validated through standard curve analysis (amplification efficiencies: 95-105%, R² > 0.99). qRT-PCR was performed on the LightCycler 96 system (Roche, Mannheim, Germany) with a 10 µL reaction volume under the following conditions: 95°C for 2 min (initial denaturation); 40 cycles of 94°C for 15 sec (denaturation) and 60°C for 30 sec (annealing/extension); followed by melting curve analysis. Three biological replicates (independent samples) and three technical replicates (PCR repeats) were included for each experimental condition. The relative expression levels were calculated using the 2^-^ΔΔCT method with β-actin as the internal reference gene.

### 2.7 Subcellular localization

The *PHB-YFP* vector was digested using *HindIII* and *SacI*, and the coding sequences of *PgGRAS48* and *PgGRAS90* were cloned into the vector (primers are listed in **Supplementary Table S1**). The recombinant plasmids were transformed into *Agrobacterium tumefaciens* GV3101 and used to infiltrate the leaves of *Nicotiana benthamiana*. YFP signals were observed using a confocal laser scanning microscope.

### 2.8 Plant materials and treatments

Wild-type *A. thaliana* (Columbia, Col-0) was purchased from Shaanxi Aiyouji Bio-technology Co., Ltd. Seeds were sterilized with 75% ethanol, rinsed with ddH_2_O, and stratified at 4°C in the dark for 3 days. They were then sown in organic-rich soil and cultivated in a growth chamber (26°C/20°C, 16 hours light/8 hours dark, relative humidity 75%) until flowering for experimental use.

### 2.9 Overexpression of *PgGRAS48* in *A. thaliana*

The *PgGRAS48* gene was cloned into the *pCAMBIA1300* vector (primers are listed in **Supplementary Table S1**) and transformed into *A. thaliana* using *Agrobacterium tumefaciens* GV3101. Seeds of wild-type (WT) and T3 generation transgenic lines were inoculated on 1/2 MS medium, and after 10 days of cultivation, the number of lateral roots, lateral root length, and main root length were recorded. Endogenous hormone levels were also measured.

### 2.10 Quantification of endogenous hormones in *A. thaliana*

The levels of GA, IAA, and 6-BA were quantified using enzyme-linked immunosorbent assay (ELISA). Plant samples were ground in liquid nitrogen and extracted with 80% methanol. After centrifugation, the supernatant was collected, dried under nitrogen gas, and resuspended in PBS. Anti-GA or anti-IAA antibodies were coated onto 96-well plates and incubated overnight at 4°C. The plates were then blocked with 1% BSA for 1 hour. Standards or samples were added and incubated at 37°C for 2 hours. After washing, HRP-conjugated secondary antibodies were added and incubated at 37°C for 1 hour. TMB substrate was added for color development, and the reaction was stopped with 2 M H_2_SO_4_. Absorbance was measured at 450 nm, and hormone concentrations were calculated.

### 2.11 The prediction of protein-protein interaction network

The 139 PgGRAS protein sequences were submitted to the STRING website, and *A. thaliana* was selected as the homologous species. After eliminating the genes that do not interact with others, the network was constructed using the genes with the highest bit score after BLAST analysis.

### 2.12 Molecular docking analysis

The three-dimensional structures of *PgGRAS48* and *PgGRAS2* were predicted using AlphaFold2 and visualized using PyMOL v.2.5.4 (https://pymol.org/2/).

### 2.13 Yeast two-hybrid assay

The full-length CDS of *PgGRAS48* was cloned into the *pGBKT7* vector, and the full-length CDS of *PgGRAS2* was cloned into the *pGADT7* vector (primers listed in **Supplementary Table S1**). The constructs were co-transformed into yeast strain AH109 and screened on SD/-Leu/-Trp/-His/-Ade and SD/-Leu/-Trp/-His/-Ade/x-α-Gal media.

### 2.14 Quantification of endogenous hormones in *A. thaliana*

For the BIFC assay, the full-length CDS of *PgGRAS2* was cloned into the *pSPYNE(R)173* vector, and the full-length CDS of *PgGRAS48* was cloned into the *pSPYCE(M)* vector (primers are provided in **Supplementary Table S1**). The two constructed vectors, along with an empty vector, were transformed into GV3101 strain. After mixing the *Agrobacterium* cultures, they were used to infect tobacco plants. YFP signals were observed under a confocal laser scanning microscope.

### 2.15 Statistical analysis

One-way ANOVA was performed using SPSS (v27.0) software to analyze the expression levels of *PgGRAS* genes in different tissues of ginseng. The root phenotype and endogenous hormone content data in *A. thaliana*, as well as the expression data of *PgGRAS* genes under different hormone treatments, were analyzed using t-tests in GraphPad Prism 8.4.2 software, and the results were visualized using this method (***p<0.001, ns not significant).

## 3 Results

### 3.1 Identification of the *GRAS* gene family in ginseng

The HMM model (PF03514) of the *GRAS* gene family was downloaded from the PFAM protein family database. Using HMMER3 software, a total of 166 protein sequences containing the GRAS domain were identified, and the conserved domains were validated through NCBI and SMART. Ultimately, 139 non-redundant *GRAS* genes were confirmed and renamed from *PgGRAS1* to *PgGRAS139* based on their chromosomal locations in ginseng (**Supplementary Table S2**). The protein characteristics of the GRAS family members in ginseng were analyzed. These proteins encode 321 (*PgGRASD114*) to 993 (*PgGRAS19*) amino acids, with molecular weights ranging from 36.20 kDa to 110.06 kDa (**Supplementary Table 2**). Among them, 134 PgGRAS proteins have an average hydrophilicity (GRAVY) score of less than 0, while only 5 PgGRAS proteins have a score greater than 0. The average isoelectric point (pI) is 4.16, indicating that most PgGRAS proteins are weakly acidic. Additionally, the predicted aliphatic index ranges from 69.77 to 102.45, indicating a high proportion of aliphatic amino acids. The instability index ranges from 35.24 to 60.27, with 9 PgGRAS proteins having an instability index greater than 40, suggesting that these proteins may be unstable and prone to denaturation or degradation. According to subcellular localization predictions, 61.15% of PgGRAS proteins are localized in nucleus, while a minority are localized in the cytoplasm, mitochondria, and other organelles.

### 3.2 Phylogenetic analysis of the *PgGRAS* gene family

**Figure 1:**
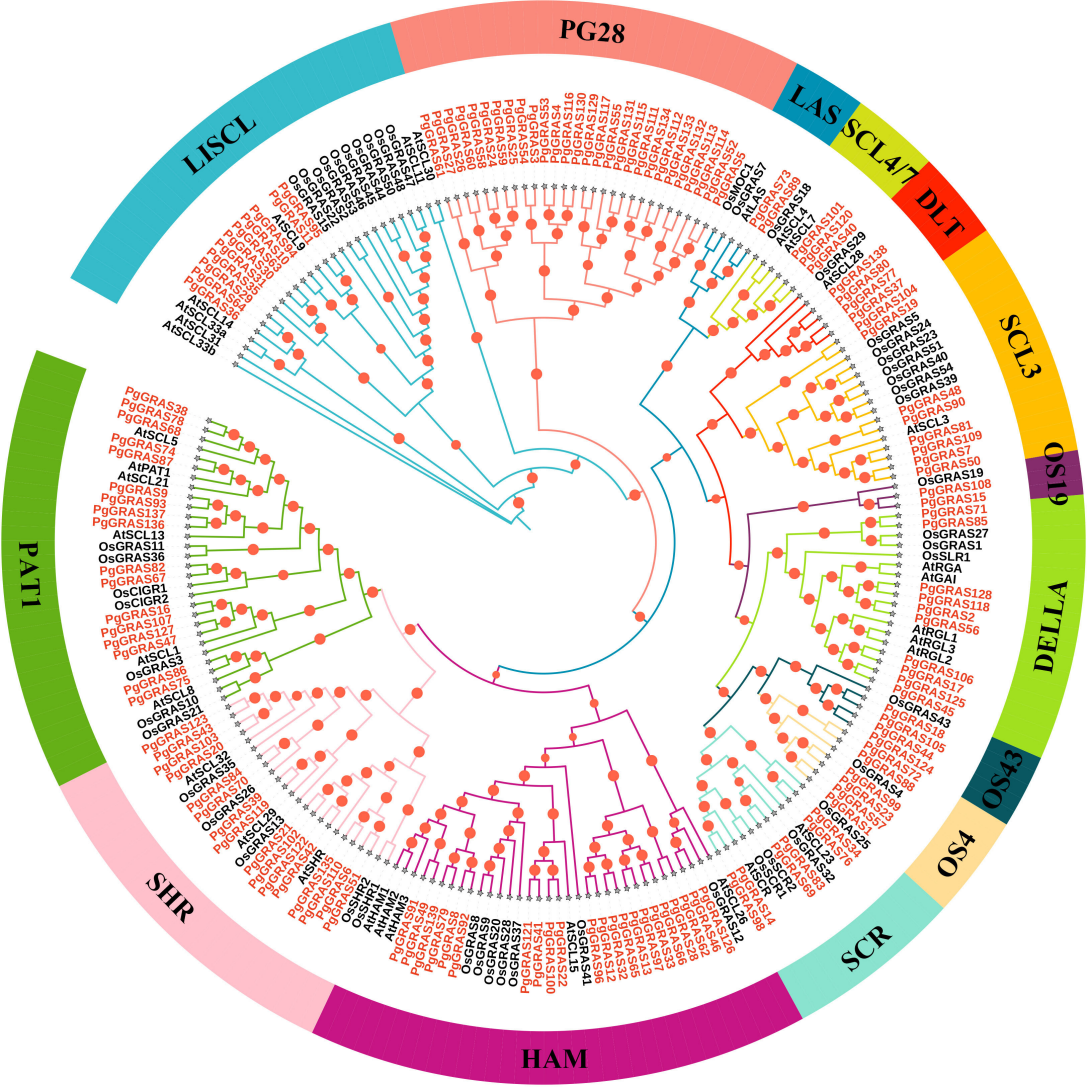
Phylogenetic tree of GRAS proteins from *P. ginseng*, *A. thaliana*, and *O. sativa*. The phylogenetic tree was constructed using the maximum likelihood method in IQ-TREE based on the JTTDCMut+F+R4 model. GRAS proteins from *Ginseng* are highlighted in red.

To further investigate the evolutionary relationships of the *GRAS* gene family in ginseng, a phylogenetic tree was constructed using IQ-TREE software, including 139 PgGRAS proteins, 33 AtGRAS proteins, and 50 OsGRAS proteins (**Supplementary Table 3**), and they were classified accordingly. The 222 GRAS proteins were divided into 14 subfamilies: LISCI, LAS, SCL4/7, DLT, SCL3, DELLA, SCR, HAM, SHR, PAT1, PG28, OS4, OS19, OS43, and PG28 (**Figure 1**). Notably, the HAM subfamily contains the largest number of members (22 PgGRAS proteins), while the OS19 subfamily has the fewest members (only 2 PgGRAS proteins) (**Figure 1**). Additionally, 28 PgGRAS proteins were found to cluster independently, not grouping with any GRAS proteins from *A. thaliana* or *O. sativa*. This subfamily was named PG28, indicating that these PgGRAS members may have undergone species-specific evolution, forming a unique gene family with potential functional or adaptive characteristics distinct from GRAS proteins in other species (**Figure 1**).

### 3.3 Gene structure and conserved motif analysis of *PgGRAS* family members

**Figure 2:**
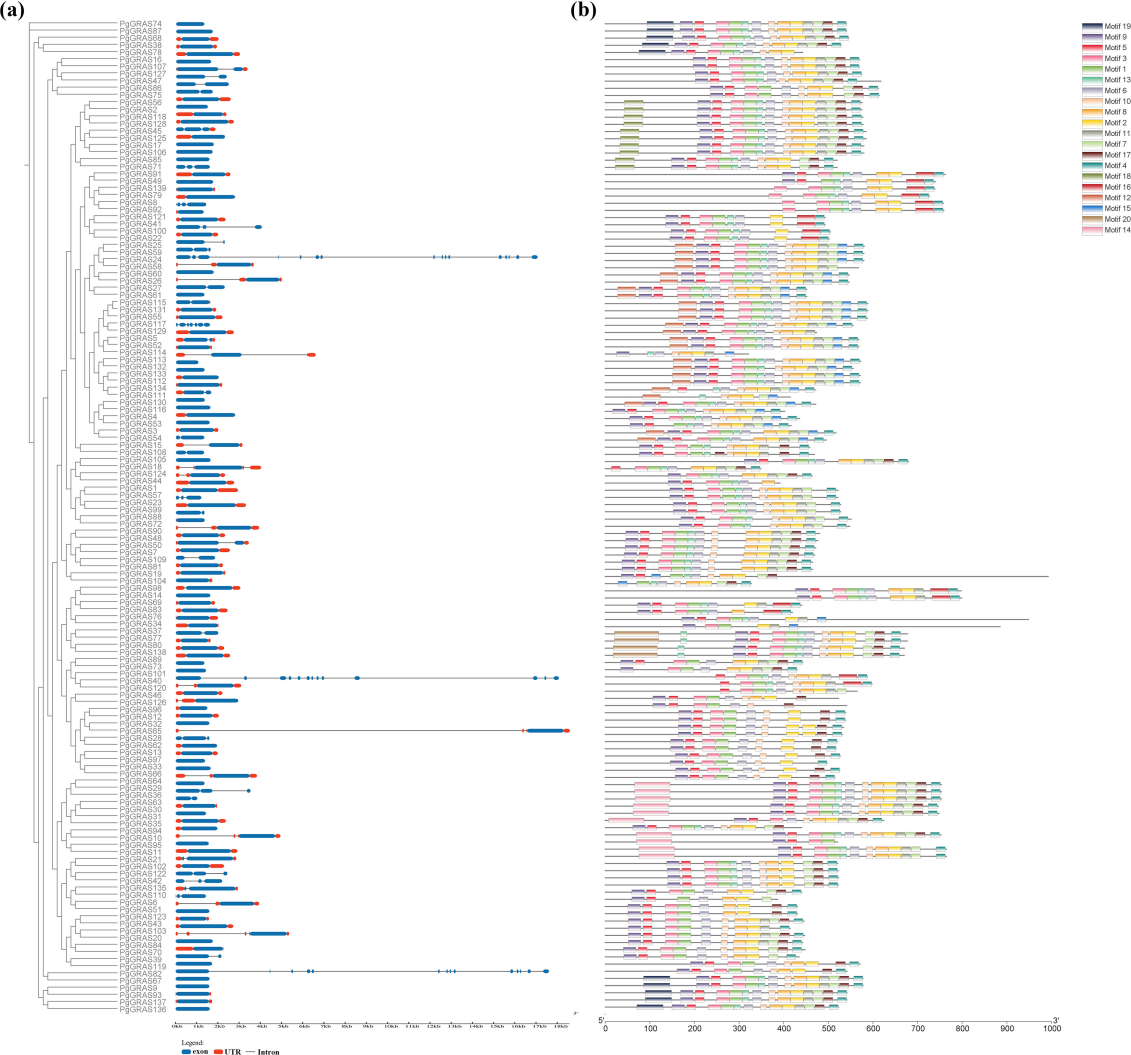
Exon-intron structure, conserved domains, and motif patterns of PgGRAS proteins (genes). **(a)** Exon-intron structure of *PgGRAS* genes. **(b)** Motif patterns of PgGRAS proteins.

To further investigate the structural characteristics of *PgGRAS* family members, the distribution of introns/exons and conserved protein motifs were analyzed (**Figure 2**). The analysis of gene structure revealed that most *PgGRAS* genes contain 1–3 exons (134 genes), followed by *PgGRAS8* (4 exons) and *PgGRAS117* (7 exons), while *PgGRAS24*, *PgGRAS82*, and PgGRAS101 contain more than 10 exons (**Figure 2a**). Additionally, 52.24% of *PgGRAS* genes contain 1–5 UTRs (**Figure 2a**). Using the MEME Suite online tool, a total of 20 conserved motifs (named Motif 1 to Motif 20) were identified. The results showed that the distribution of motifs in the C-terminal region was significantly more abundant than in the N-terminal region (**Figure 2b**; **Supplementary Figure 1**). All PgGRAS protein members contain the corresponding conserved GRAS domain. The core part of the GRAS domain, the VHIID domain, is located in Motif 1, while Motif 3, 5, 9 and Motif 6, 13 are located in the LHRI and LHRII domains flanking the VHIID domain, respectively. This is followed by the PFYRE domain in Motif 2 and the SAM domain in the C-terminal region within Motif 4 (**Figure 2b**). Members of the same subgroup exhibit subfamily-specific motif patterns. For example, all 10 PgGRAS members in the DELLA subfamily contain the unique Motif 18, and the DLT subfamily contains the unique Motif 20 (**Figure 2b**). Additionally, Motif 12 is exclusively present in the PG28 subfamily, Motif 9 is unique to the PAT1 subfamily, and Motif 14 is located only at the N-terminus of LISCL subfamily members (**Figure 2b**). Overall, the diverse structural features observed across subfamilies reflect their broad functional diversity.

### 3.4 *PgGRAS* genes cis-acting element prediction and transcriptional regulatory network analysis

Cis-acting elements, as non-coding DNA regions in gene promoters, can regulate the transcriptional activity of their linked genes. The distribution of cis-acting elements in promoters may be closely related to the functional diversity and expression pattern complexity of genes (Lescot et al., 2002). By analyzing the 1.5 kb upstream sequences of *PgGRAS* gene promoters and identifying them using the PlantCARE database, a total of 55 types of cis-acting elements were identified (**Supplementary Figure 2**, **Supplementary Table 3**). Light-responsive elements were universally present in the promoter regions of all *PgGRAS* genes and dominated in quantity, accounting for 51% of the total elements. It was found that 29 *PgGRAS* genes contain 9 types of elements related to plant hormone responses, including methyl jasmonate-responsive elements (CGTCA-motif, TGACG-motif), salicylic acid-responsive elements (TCA-element), abscisic acid-responsive elements (ABRE), gibberellin-responsive elements (GARE-motif, P-box, TATC-box), and auxin-responsive elements (TGA-element, AuxRR-core) (**Supplementary Figure 2**). In the promoter regions of *PgGRAS* genes, 7 types of cis-acting elements were identified to be involved in regulating the development of different tissues in ginseng, such as meristems, endosperm, and seeds (**Supplementary Figure 2**). Additionally, 42% of the genes contain elements related to stress responses, including ARE (anaerobic response element), LTR (low-temperature response element), MBS (drought response element), TC-rich repeats (stress response element), and GC-motif (stress response element) (**Supplementary Figure 2**). This indicates that *PgGRAS* genes not only participate in tissue development but also play important roles in responding to various abiotic stresses.

### 3.5 Collinearity analysis of the *PgGRAS g*ene family

**Figure 3:**
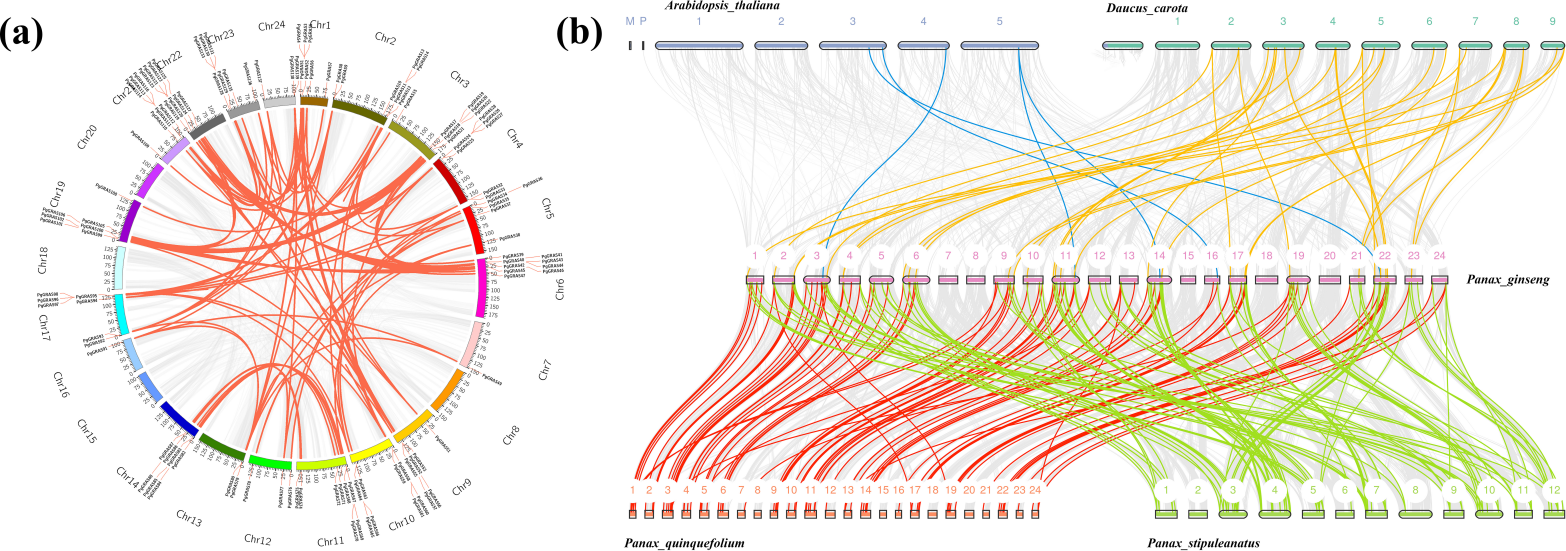
Chromosomal distribution and evolutionary relationship analysis of *PgGRAS* gene family members in *Ginseng*. **(a)** Intraspecies collinearity of *PgGRAS* genes. Colored blocks represent parts of *Ginseng* chromosomes. Red lines indicate duplicated *PgGRAS* gene pairs. **(b)** Interspecies collinearity between *Panax ginseng* and *Arabidopsis thaliana*, *Daucus carota*, *Panax quinquefolium*, and *Panax stipuleanatus*. Different colors represent duplicated *PgGRAS* pairs between *Ginseng* and different species.

Chromosomal localization analysis revealed that the 139 *PgGRAS* genes are unevenly distributed across 20 chromosomes of the ginseng genome (out of 24 chromosomes). Chromosomes 4, 19, and 21 contain the highest number of *PgGRAS* genes (13.9%, 10 genes each), while the lowest numbers were observed on chromosomes 7 and 16 (1.44%, 2 genes each) (**Figure 3a**). Gene duplication plays a crucial role in functional innovation and gene family expansion. Therefore, a detailed analysis of *PgGRAS* gene duplication events in the ginseng genome was conducted. The results showed that 130 pairs of *PgGRAS* genes exhibit collinearity, with 11 pairs originating from tandem duplication events located on chromosomes 1, 2, 4, 10, 17, 21, and 23, while the remaining 119 pairs arose from segmental duplication events (**Figure 3a**). Interspecies collinearity analysis indicated that 120 *PgGRAS* genes have homologous relationships with genes from multiple plant species, including *Arabidopsis thaliana* (5 pairs), *Daucus carota* (34 pairs), *Panax quinquefolium* (91 pairs), and *Panax stipuleanatus* (116 pairs) (**Figure 3b**). Notably, the proportion of orthologous gene pairs between ginseng and its closely related species, *Panax quinquefolium* and *Panax stipuleanatus*, was significantly higher, reaching 65.47% and 83.45%, respectively. Additionally, *PgGRAS9/14/23/80/93/99/123/138* were associated with at least 4 homologous gene pairs, suggesting that these genes may have played an important role in the evolutionary expansion of the *GRAS* gene family (**Figure 3b**).

To investigate the evolutionary patterns and selection pressure of *PgGRAS* genes, Ka/Ks analysis was performed on duplicated *PgGRAS* gene pairs. The results showed that the Ka values of 127 *PgGRAS* gene pairs ranged from 0.0024 to 0.441, while the Ks values ranged from 0.011 to 2.294 (**Supplementary Table S5**). Further analysis revealed that the Ka/Ks ratios ranged from 0.047 to 1.103, with only one homologous gene pair (*PgGRAS21-PgGRAS102*) having a Ka/Ks ratio greater than 1, indicating that they may have undergone positive selection. The Ka/Ks ratios of the remaining duplicated genes were all less than 1 (**Supplementary Table S5**). These results suggest that the *PgGRAS* gene family primarily maintained functional stability through purifying selection during evolution, while individual gene pairs may have acquired new adaptive functions through positive selection.

### 3.6 Differential expression analysis of *PgGRAS* genes in different tissues and under hormone treatments

**Figure 4:**
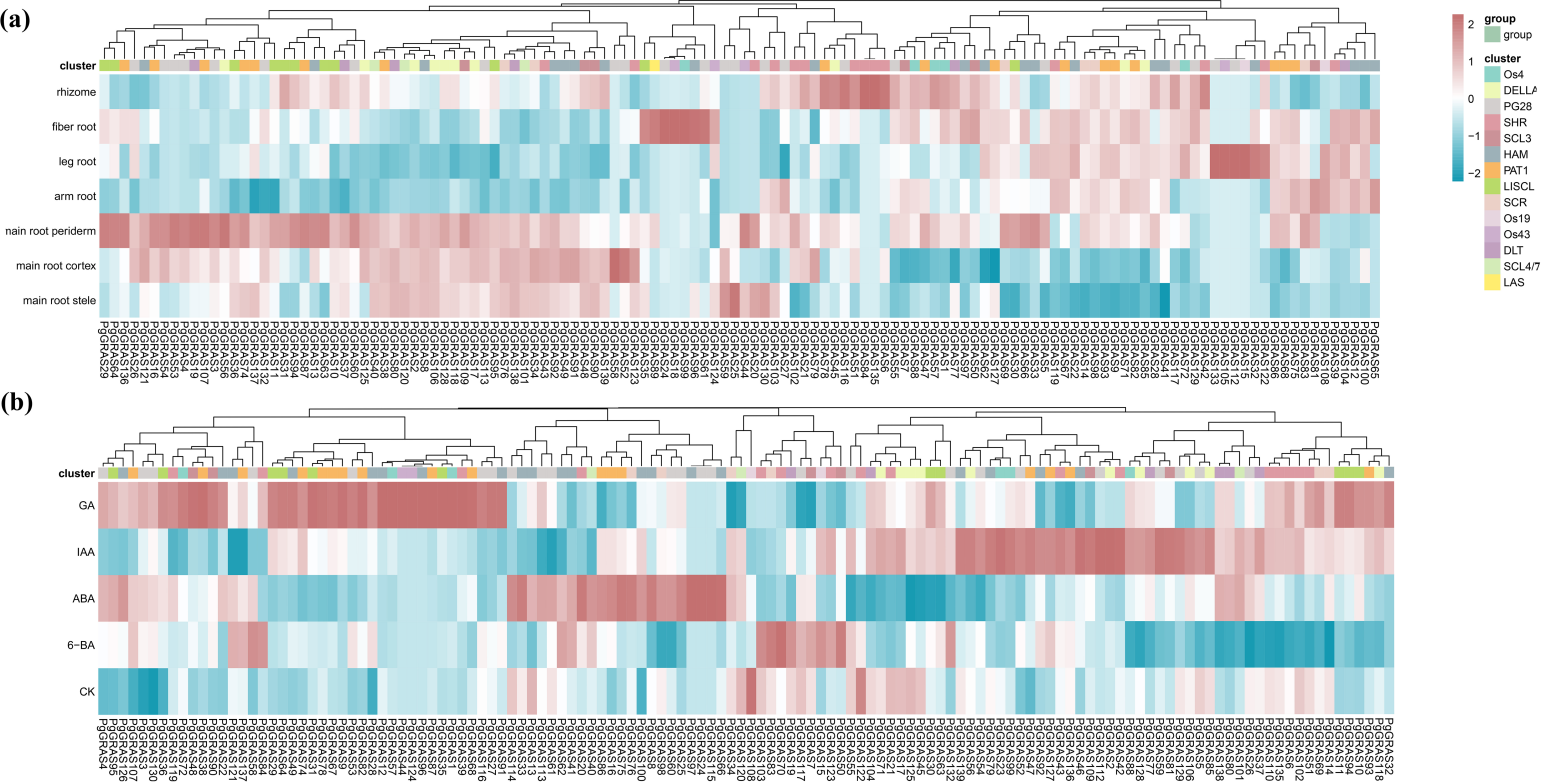
Expression patterns of *PgGRAS* genes. **(a)** Expression levels of *PgGRAS* genes in different tissues. **(b)** Expression levels of *PgGRAS* genes under different hormone treatments.

**Figure 5:**
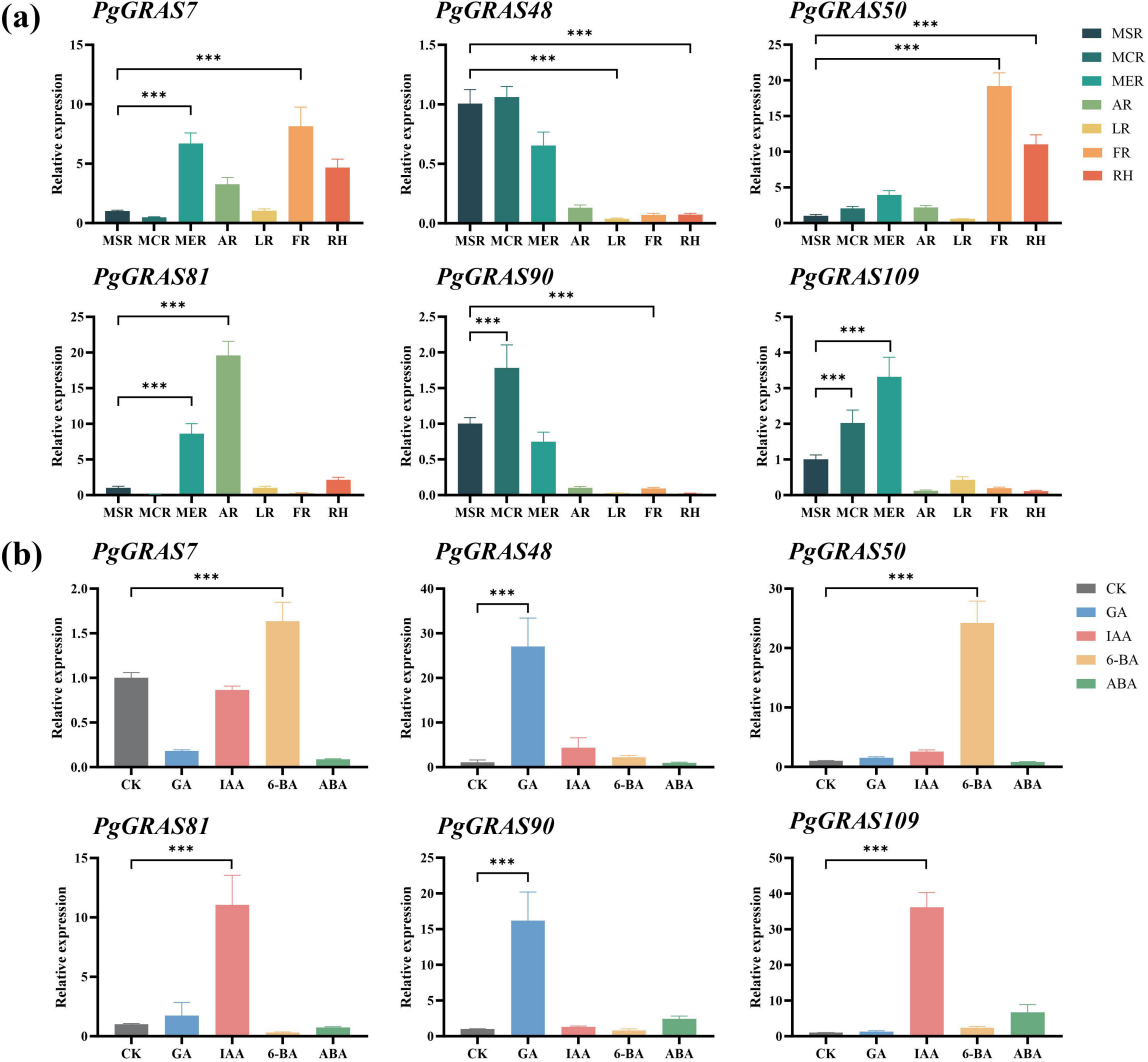
RT-qPCR analysis of *PgGRAS* expression in *Ginseng* under different tissue locations and hormone treatments. **(a)** Expression levels of *PgGRAS* in different tissue locations (n = 3). **(b)** Expression levels of *PgGRAS* under different hormone treatments (n =3). main root periderm (MER), main root cortex (MCR), main root stele (MSR), rhizome (RH), leg root (LR), fiber root (FR), and arm root (AR). Statistical significance is indicated by asterisks: ***p<0.001..

By analyzing published transcriptome data, we revealed the expression characteristics of *PgGRAS* genes in different root tissues and under hormone treatment conditions (**Figure 4**). The results showed that *PgGRAS* members of the PAT1, SCL3, and SCR subfamilies were highly expressed in various root tissues (main root periderm, main root cortex, main root stele, leg root, fibrous root, arm root, and rhizome), while *PgGRAS* genes in the LISCL subfamily exhibited significantly higher expression in the main root periderm compared to other root tissues (**Figure 4a**). Additionally, some *PgGRAS* genes showed organ-specific expression in the root, with *PgGRAS58* expressed exclusively in the main root cortex and *PgGRAS18* expressed only in fibrous root (**Figure 4a**). This tissue-specific expression pattern suggests that these genes may play specific biological roles in different root regions. Furthermore, the expression patterns of *PgGRAS* genes exhibited significant differences under various hormone treatments. *PgGRAS* genes in the LISCL subfamily showed enhanced expression levels after IAA treatment, while most PgGRAS genes in the PAT1 subfamily (*PgGRAS9/38/67/74/82/87/93*) were significantly upregulated after GA treatment (**Figure 4b**). Additionally, it was found that *PgGRAS24*, *PgGRAS44*, and *PgGRAS96* genes specifically responded to GA, while *PgGRAS24*, *PgGRAS97*, and *PgGRAS115* genes specifically responded to IAA (**Figure 4b**). The relative expression levels of six SCL3 subfamily genes in root tissues and under hormone treatments were evaluated using qRT-PCR (**Figure 5**). The results showed that *PgGRAS48*, *PgGRAS90*, and *PgGRAS109* had higher expression levels in the main root periderm, main root cortex, and main root stele, while *PgGRAS7* and *PgGRAS50* were highly expressed in fibrous root and rhizome, and *PgGRAS81* exhibited higher expression in the main root periderm and arm root (**Figure 5a**). The expression profiles of SCL3 subfamily members under hormone treatments showed different trends. *PgGRAS48* and *PgGRAS90* were significantly upregulated under GA induction, *PgGRAS81* and Pg*GRAS109* had higher expression levels after IAA treatment, and *PgGRAS7* and *PgGRAS50* responded more prominently to 6-BA (**Figure 5b**). These results support the expression patterns observed in the transcriptome data, indicating the high reliability of the transcriptome data.

### 3.7 Functional and interaction studies of the *PgGRAS48 g*ene

**Figure 6:**
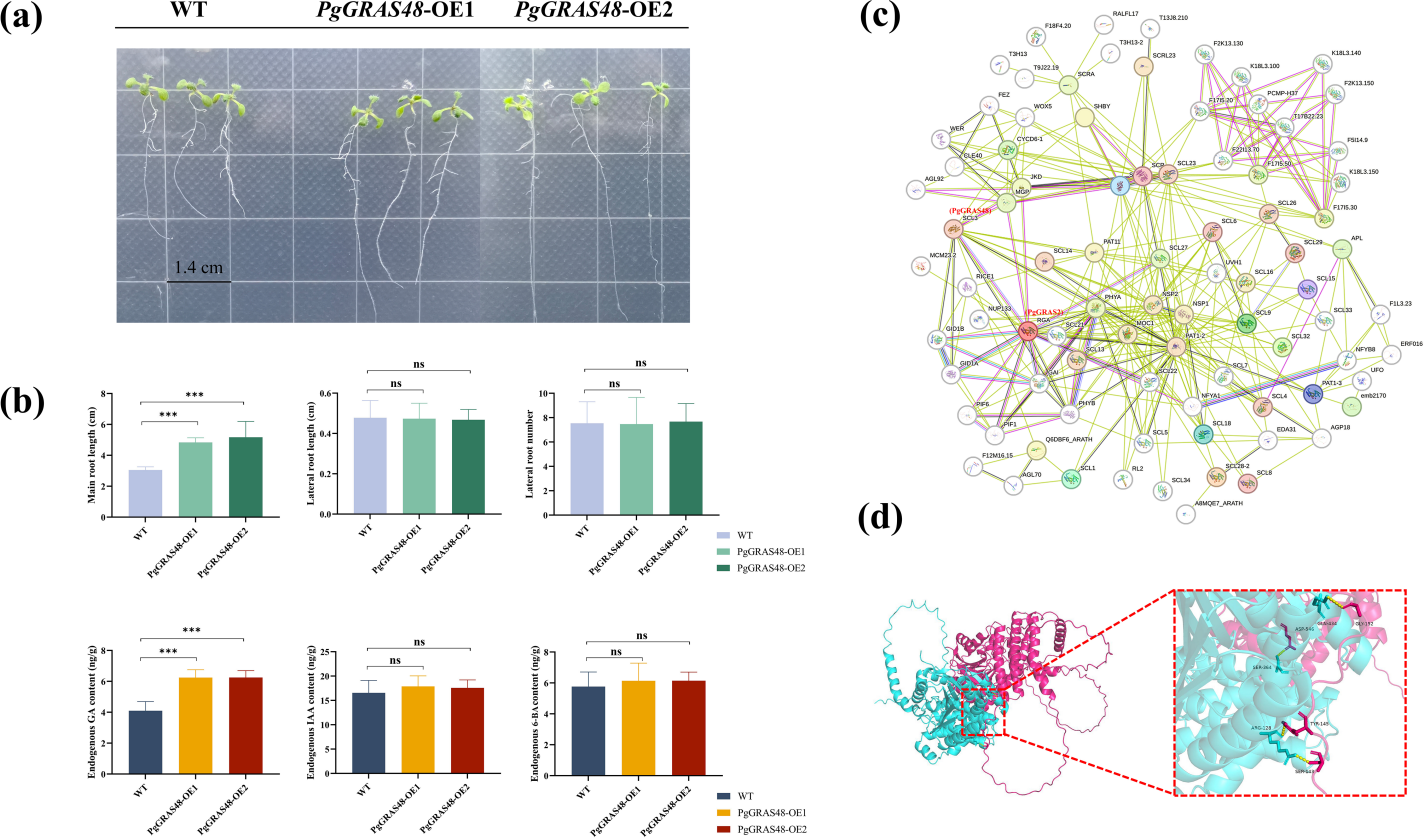
Functional analysis of PgGRAS48 transgenic *A*. *thaliana* and prediction of protein-protein interactions. **(a)** Phenotypes of wild-type (WT) and *PgGRAS48* overexpression lines at 2 weeks old. Scale bar: 1.4 cm. **(b)** Root phenotypes and endogenous hormone content statistics of WT and *PgGRAS48* transgenic plants. Error bars represent standard deviation (SD; n = 3). **(c)** Protein-protein interaction prediction of PgGRAS proteins. **(d)** Molecular docking analysis of the interaction between PgGRAS48 and PgGRAS2 proteins. Statistical significance is indicated by asterisks: ***p<0.001, ns, not significant.

**Figure 7:**
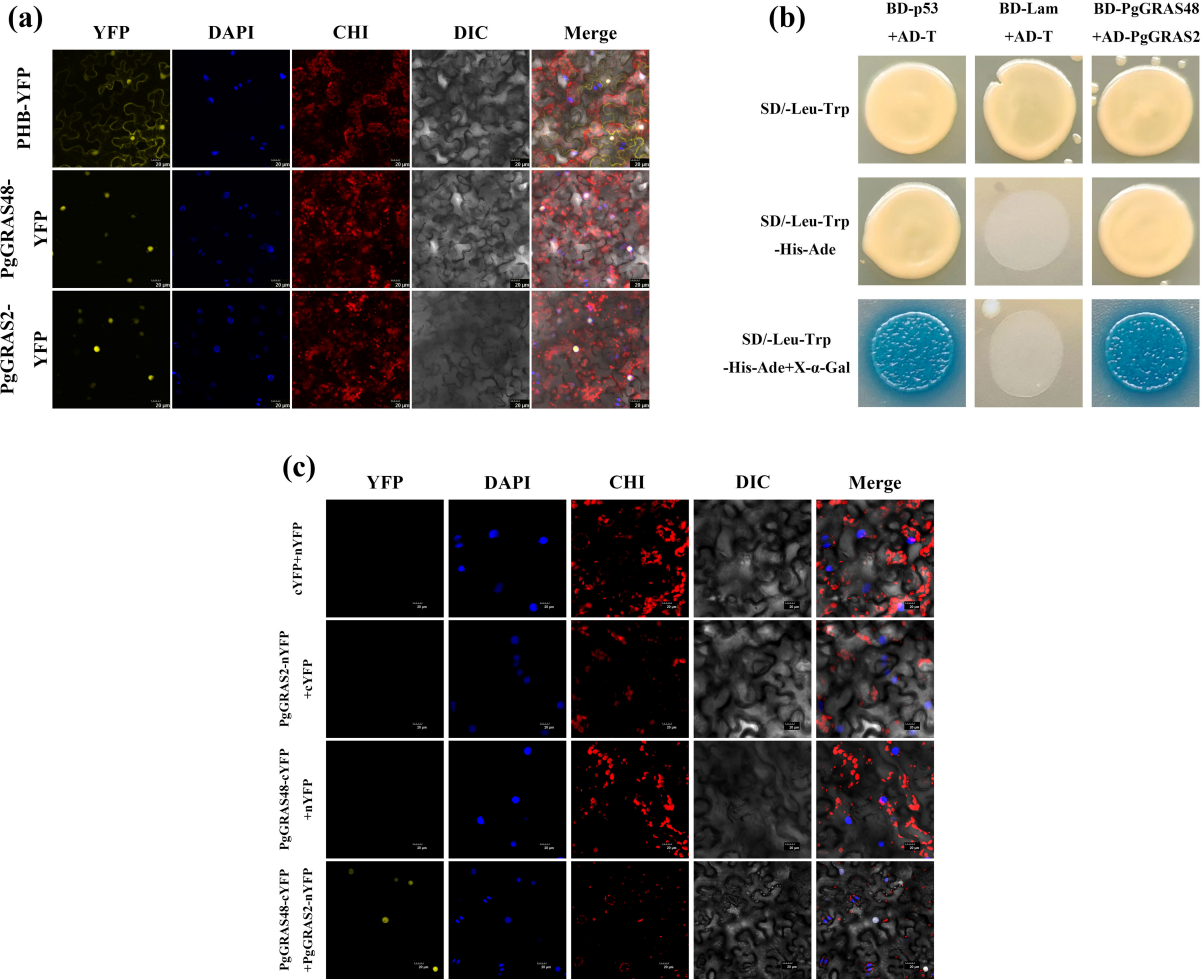
Subcellular localization of *PgGRAS48* and *PgGRAS2* and their interaction validation. **(a)** Subcellular localization analysis of PgGRAS2 and PgGRAS48 in *Nicotiana tabacum* of leaves. **(b)** Yeast two-hybrid validation of the interaction between PgGRAS48 and PgGRAS2. **(c)** BIFC analysis.

To reveal the key regulatory role of the *PgGRAS48* gene in plant root development, this study constructed *PgGRAS48* overexpression lines. The identified positive transgenic plants were named *PgGRAS48*-OE1 and *PgGRAS48*-OE2 (**Figure 6a**). Statistical analysis of root phenotypes showed that the main root lengths of overexpression lines OE-1 and OE-2 were significantly longer than those of the wild-type (WT), while the lateral root lengths and numbers showed no significant changes (**Figure 6b**). Additionally, the endogenous GA content in the main roots of overexpression *A. thaliana* lines was significantly higher than that of WT, with no significant changes in endogenous IAA or 6-BA levels (**Figure 6b**). Protein-protein interaction analysis and AlphaFold2-based protein structure prediction revealed that PgGRAS48 and PgGRAS2 interact through specific binding between four pairs of key amino acid residues, forming a stable heterodimeric complex (**Figures 6c, d**). To investigate their subcellular localization, we constructed two fusion expression vectors (35S-PgGRAS48-YFP and 35S-PgGRAS2-YFP). Fluorescence microscopy showed that while YFP signals in the control group were diffusely distributed across both the cell membrane and nucleus, the 35S-PgGRAS48-YFP and 35S-PgGRAS2-YFP fusion proteins exhibited predominant nuclear localization (**Figure 7a**). Subsequently, the protein interaction between PgGRAS48 and PgGRAS2 was validated using Y2H assays. Specifically, the *PgGRAS48* gene was cloned into the pGBKT7 vector to construct the bait protein, while the *PgGRAS2* gene was inserted into the pGADT7 vector to construct the prey protein. Screening on quadruple dropout medium (SD/-Leu/-Trp/-His/-Ade) supplemented with X-α-Gal and Aba showed that yeast colonies co-transformed with PgGRAS48-BD and PgGRAS2-AD turned blue (**Figure 7b**). The BiFC assay further confirmed the specific binding between PgGRAS48 and PgGRAS2 (**Figure 7c**), demonstrating their direct interaction.

## 4 Discussion

The GRAS gene family plays a pivotal role in regulating the progression of plant growth and development (Laskar et al., 2021), such as the response to GA signaling and establishing radial root patterning and phytohormone signaling pathways (Guo et al., 2017). However, the widespread distribution and functional diversity of *GRAS* family members in ginseng have not been systematically studied. In this study, we conducted a comprehensive analysis of the *GRAS* gene family in ginseng, delving into the phylogenetic relationships, gene structures, chromosomal localization, cis-acting elements, and expression patterns of *PgGRAS* genes. A total of 139 *PgGRAS* genes were identified at the genome level of ginseng (**Supplementary Table 1**), a number significantly higher than those in *Arabidopsis thaliana* (34) (Liu and Widmer, 2014), *O. sativa* (60) (Liu and Widmer, 2014), *Secale cereale* (67) (Fan et al., 2024), *Liriodendron chinense* (49) (Weng et al., 2023), *Dendrobium chrysotoxum* (46) (Zhao et al., 2022), *Hibiscus hamabo* (59) (Ni et al., 2022), and *Castanea mollissima* (48) (Yu et al., 2022). Such differences in *GRAS* gene numbers among species may be closely related to gene duplication events or variations in genome size (Grimplet et al., 2016). As an allotetraploid, ginseng shows polyploidy-associated lineage-specific expansion of *GRAS* transcription factors, likely contributing to functional diversification of duplicated genes and increased regulatory complexity in development and stress responses (Li et al., 2021). To clarify the evolutionary relationships, classification, and functional conservation of *PgGRAS* genes, a phylogenetic tree containing *AtGRAS* and *OsGRAS* genes was constructed. The results showed that the 139 *PgGRAS* genes can be divided into 14 subfamilies: LISCL, LAS, SCL4/7, DLT, SCL3, DELLA, SCR, HAM, SHR, PAT1, OS4, OS19, OS43, and PG28 (**Figure 1**). Additionally, 28 *PgGRAS* genes were found to cluster independently and were named PG28, suggesting that these genes may have undergone ginseng-specific evolution or functional differentiation, potentially related to the unique biological characteristics of ginseng. The remaining 13 subfamilies of *PgGRAS* proteins were grouped into the same branches as *A. thaliana* and *O. sativa*, indicating high conservation during evolution and their potential involvement in fundamental physiological processes common to plants.

Our analysis revealed that over half of *PgGRAS* genes are intronless (**Figure 2a**), mirroring the high frequency observed in *Solanum lycopersico*n (77.4%) (Huang et al., 2015) and *Prunus mume* (82.2%) (Lu et al., 2015), while three members (*PgGRAS24/82/101*) contain more than 10 exons, suggesting evolutionary intron loss and gene fragment insertion events (Rogozin et al., 2012; Staiger and Brown, 2013). Typical GRAS proteins contain highly conserved domains at the C-terminus, primarily including the VHIID motif and its flanking LRI and LRII regions, as well as the PFYRE, RVER, and SAW motifs (Khan et al., 2022). These domains are hallmark features of the GRAS family proteins and are widely involved in protein-protein and protein-nucleic acid interactions. Analysis of conserved domains in PgGRAS proteins revealed that PgGRAS31, PgGRAS111, PgGRAS114, and PgGRAS134 lack conserved histidine and aspartic acid residues in the VHIID region, while the remaining VHIID regions exhibit many variations in histidine and aspartic acid residues (**Figure 2b**), a phenomenon also observed in Secale cereale (Fan et al., 2024). These variations in the VHIID domain challenge the traditional notion of its absolute conservation among GRAS proteins, suggesting that domain diversification can facilitate the formation of novel regulatory partnerships while preserving core GRAS functionality (Khan et al., 2022). This hypothesis aligns with recent findings in *A. thaliana*, where mutations in key residues of the VHIID domain led to specific alterations in the GA signaling pathway (Yoshida and Ueguchi-Tanaka, 2014), and is consistent with studies on the *O. sativa* DELLA protein SLR1, which demonstrated that structural reorganization of the VHIID domain modulates gibberellin perception (Itoh, 2002). Additionally, members within the same subfamily share similar conserved motifs. The DELLA subfamily uniquely contains Motif18, the DLT subfamily specifically includes Motif20, while Motif12, Motif9 and Motif14 are exclusively present in the PG28, PAT1 and LISCL subfamilies, respectively (**Figure 2b**). This subfamily-specific motif distribution suggests these conserved domains may confer specialized functional properties that contribute to the distinct regulatory roles of each *PgGRAS* subfamily in plant development and stress responses.

Gene duplication is a core mechanism driving gene family expansion and functional innovation, playing a key role in genome evolution, environmental adaptation, and the formation of new species (Fang et al., 2022). Through intraspecies collinearity analysis in ginseng, we identified 130 pairs of *PgGRAS* genes with collinear relationships (**Figure 3a**). Among these, segmental duplication events were the primary driver of gene family expansion, accounting for over 91%, while tandem duplication events were relatively rare. These duplication events may lead to functional redundancy and gene diversification, providing a genetic basis for species adaptation to complex environments (Clark, 2023). Further comparison of genomic collinearity between ginseng and species such as *Arabidopsis thaliana*, *Daucus carota*, *Panax quinquefolium*, and *Panax stipuleanatus* revealed that these genes are highly conserved within the *Panax genus* but exhibit less collinearity with *Arabidopsis thaliana* and *Daucus carota* (**Figure 3b**), suggesting that these genes may have been newly acquired during the evolution of *Panax* species, reflecting species-specific functional differentiation (Freschet et al., 2021). Ka/Ks ratio analysis showed that the vast majority of duplicated gene pairs (126 pairs) had Ka/Ks values less than 1, indicating that they were subject to purifying selection during evolution and are highly conserved in function. Only one gene pair had a Ka/Ks value greater than 1, suggesting it may have undergone positive selection (**Supplementary Table S5**). This may be related to ginseng’s environmental adaptation or the acquisition of specific functions. These findings provide important insights into the evolutionary history of the ginseng gene family and its role in species adaptation.

In this study, by integrating transcriptome data and qRT-PCR validation, we systematically analyzed the potential roles of the *PgGRAS* gene family in tissue-specific expression and hormone responses in roots. Transcriptome analysis revealed that most SCL3 subfamily members are highly expressed in the cortex, periderm, and stele of the main root (**Figure 4a**). qRT-PCR validation of selected SCL3 subfamily genes further supported the reliability of the transcriptome data (**Figure 5a**), a pattern consistent with reports in *A. thaliana* and *O. sativa*, where SCL3 homologs regulate root meristem activity via GA signaling (Neves et al., 2023). Notably, *PgGRAS48* and *PgGRAS90* exhibited GA-specific induction (**Figures 4b**), with their expression levels being significantly upregulated by GA treatment (**Figure 5b**). This GA responsiveness mirrors the conserved function of SCL3 homologs like *AtSCL3* in *A. thaliana*, which antagonizes DELLA proteins to activate GA signaling (Seemann et al., 2022). However, unlike *AtSCL3* that predominantly integrates GA and auxin pathways, *PgGRAS48* uniquely retained GA specificity while other SCL3 members (*e.g., PgGRAS7/50*) showed broader responsiveness to cytokinin (6-BA) or auxin (IAA). This functional difference is similar to the findings in *O. sativa* - *OsGRAS23* regulates lateral root development through cytokinin signaling (Xu et al., 2015; Wang et al., 2024). However, *PgGRAS48* exhibits unique characteristics: its specific high expression in the cortex, periderm, and stele of main roots, and strict responsiveness to GA signals, suggesting that it drives main root elongation through the GA pathway in these key tissues rather than participating in multi-hormone interaction processes.

In this study, by constructing *PgGRAS48* overexpression lines and combining phenotypic analysis, we revealed its key role in regulating root elongation. Experimental results demonstrated that overexpression of *PgGRAS48* significantly promoted main root elongation, accompanied by a marked increase in endogenous GA content within the main roots (**Figures 6a-b**). This finding aligns with the canonical role of SCL3 subfamily members in GA signaling, where SCL3 likely indirectly regulates the expression of GA biosynthetic genes by promoting GA signal transduction, thereby maintaining GA homeostasis (Zhang et al., 2011). Subsequently, through protein-protein interaction analysis and AlphaFold2 prediction, we identified an interaction between PgGRAS48 and PgGRAS2, a member of the DELLA subfamily (**Figures 6c-d**). Previous studies have demonstrated the diversity of DELLA-SCL3 interaction patterns: in *A. thaliana*, these proteins form an indirect complex through INDETERMINATE DOMAIN (IDD) family proteins to coordinately regulate GA signaling feedback pathways, where DELLA represses target gene expression while SCL3 antagonizes DELLA to release GA signals (Yoshida and Ueguchi-Tanaka, 2014). However, our study provides the first experimental evidence through BiFC and yeast two-hybrid assays that PgGRAS48 (SCL3) and PgGRAS2 (DELLA) can directly interact without requiring IDD scaffold proteins (**Figures 7b-c**). This finding challenges the conventional model of exclusively IDD-mediated SCL3-DELLA interactions and suggests a dual regulatory mechanism - direct interaction may serve as a rapid response system (e.g., for immediate root development regulation), while IDD-mediated complexes maintain long-term GA homeostasis. Furthermore, *PgGRAS48* overexpression enhances GA biosynthesis capacity (**Figure 6b**). Recent studies indicate that DELLA can regulate chromatin accessibility by binding to the SWI/SNF complex core subunit SWI3C (Sarnowska et al., 2013). Combined with our results, we propose that PgGRAS48 may recruit the SWI/SNF complex to GA biosynthetic gene loci through its interaction with DELLA, cooperatively opening chromatin structure to establish an “SCL3-DELLA-SWI/SNF” ternary regulatory model. Notably, while SCL3-DELLA interactions primarily regulate hypocotyl elongation in *A. thaliana* (Waseem et al., 2022), our study reveals that *PgGRAS48* overexpression significantly promotes main root elongation (**Figure 6a**), suggesting species- or tissue-specific regulatory divergence. This functional difference may stem from the ginseng SCL3-DELLA module specifically activating root development-related target genes (e.g., *EXPANSINs*) (Samalova et al., 2023), with PgSCL3 showing high root-specific expression patterns, indicating its unique regulatory role in root morphogenesis. Future studies should focus on resolving the precise molecular interface of SCL3-DELLA interactions and elucidating how they coordinate GA metabolism with root tissue-specific development through downstream target gene networks.

## 5 Conclusions

In this study, we systematically identified 139 *GRAS* gene family members (*PgGRAS*) in the *Ginseng* genome for the first time and classified them into 14 subfamilies, including a newly discovered species-specific subfamily, PG28. Through gene structure, conserved motif, and phylogenetic analyses, we revealed the functional differentiation and evolutionary conservation of different subfamilies. Collinearity analysis indicated that segmental duplication is the primary driver of the expansion of the *GRAS* family in ginseng. Experimental studies have demonstrated that the overexpression of *PgGRAS48* significantly promotes root growth in *A. thaliana* by elevating endogenous gibberellin (GA) levels. Further molecular interaction analyses confirmed that *PgGRAS48* can directly bind to *PgGRAS2* (a member of the DELLA subfamily), suggesting its potential role in regulating GA biosynthesis or signaling pathways. These findings not only expand our understanding of the functional diversity within the *PgGRAS* gene family but also reveal the existence of the novel PG28 subfamily and lineage-specific expansion of *GRAS* genes in ginseng, reflecting genetic innovation during its evolution. Notably, *PgGRAS48* may regulate GA biosynthesis and signaling pathways through the SCL3-DELLA protein interaction network, thereby promoting main root elongation. However, its precise regulatory network and molecular mechanisms remain to be elucidated. Future research should prioritize the use of ginseng CRISPR gene-editing technology to validate the native function of *PgGRAS48* and decipher its molecular mechanisms, while also exploring the adaptive evolutionary significance of the PG28 subfamily. Integrating multi-omics approaches will provide a theoretical foundation for the genetic improvement of medicinal root traits.

## Data Availability

Publicly available datasets were analyzed in this study. This data can be found here: https://www.cncb.ac.cn/.
