# Supplementary material for: Genome-wide identification of the GRAS gene family and evidence for the involvement of PgGRAS48 in main root development in Panax ginseng
Source: Front Plant Sci. 2025 Jun 13;16:1603268. doi: 10.3389/fpls.2025.1603268 (PMC12202484; doi:10.3389/fpls.2025.1603268)
Supplement: Supplementary file 1 [file DataSheet1.docx]

Supplementary Material

## Supplementary Figures


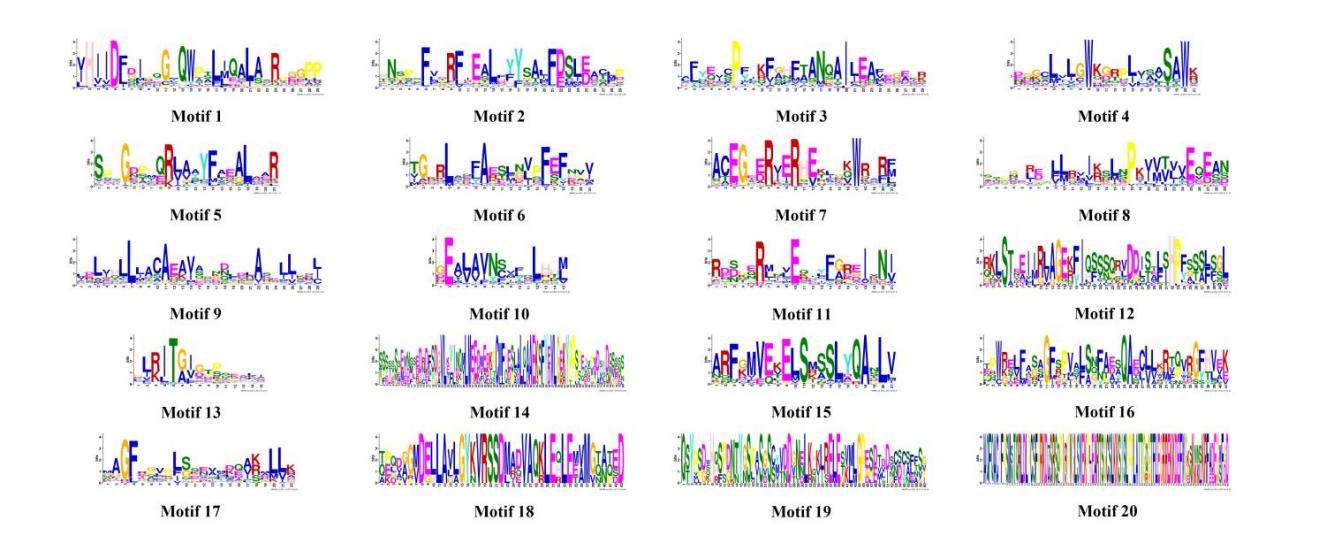


**Supplementary figure 1.** Prediction of 20 conserved motifs in PgGRAS by MEME analysis.


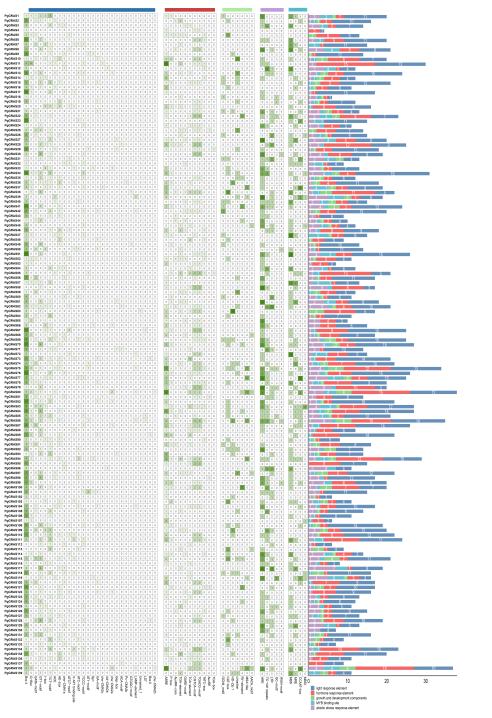


**Supplementary figure 2.** Cis-acting regulatory elements analysis within 1500 bp upstream regions of PgGRAS genes.
